# Community IntraVenous Antibiotic Study (CIVAS): protocol for an evaluation of patient preferences for and cost-effectiveness of community intravenous antibiotic services

**DOI:** 10.1136/bmjopen-2015-008965

**Published:** 2015-08-21

**Authors:** C Czoski Murray, M Twiddy, D Meads, S Hess, J Wright, E D Mitchell, C Hulme, S Dodd, H Gent, A Gregson, K McLintock, D K Raynor, K Reynard, P Stanley, R Vincent, J Minton

**Affiliations:** 1 Leeds Institute of Health Sciences, University of Leeds, Leeds, UK; 2 Institute for Transport Studies, University of Leeds, Leeds, UK; 3 Leeds Teaching Hospitals NHS Trust, Leeds, UK; 4 Service user; 5 Leeds Community Healthcare Trust, Leeds, UK; 6 School of Healthcare, University of Leeds, Leeds, UK; 7 Bradford Teaching Hospitals NHS Foundation Trust, Leeds, UK

**Keywords:** HEALTH ECONOMICS, INFECTIOUS DISEASES, QUALITATIVE RESEARCH

## Abstract

**Introduction:**

Outpatient parenteral antimicrobial therapy (OPAT) is used to treat a wide range of infections, and is common practice in countries such as the USA and Australia. In the UK, national guidelines (standards of care) for OPAT services have been developed to act as a benchmark for clinical monitoring and quality. However, the availability of OPAT services in the UK is still patchy and until quite recently was available only in specialist centres. Over time, National Health Service (NHS) Trusts have developed OPAT services in response to local needs, which has resulted in different service configurations and models of care. However, there has been no robust examination comparing the cost-effectiveness of each service type, or any systematic examination of patient preferences for services on which to base any business case decision.

**Methods and analysis:**

The study will use a mixed methods approach, to evaluate patient preferences for and the cost-effectiveness of OPAT service models. The study includes seven NHS Trusts located in four counties. There are five inter-related work packages: a systematic review of the published research on the safety, efficacy and cost-effectiveness of intravenous antibiotic delivery services; a qualitative study to explore existing OPAT services and perceived barriers to future development; an economic model to estimate the comparative value of four different community intravenous antibiotic services; a discrete choice experiment to assess patient preferences for services, and an expert panel to agree which service models may constitute the optimal service model(s) of community intravenous antibiotics delivery.

**Ethics and dissemination:**

The study has been approved by the NRES Committee, South West—Frenchay using the Proportionate Review Service (ref 13/SW/0060). The results of the study will be disseminated at national and international conferences, and in international journals.

**Strengths and limitations of this study**

- A key outcome of this study will be an understanding of patient preferences for services.
- An economic model evaluating the value-for-money of outpatient parenteral antimicrobial therapy (OPAT) services.
- The potential beneficiaries of the research are National Health Service (NHS) Trusts and those responsible for commissioning services as this will provide a robust evidence base on which to make service provision decisions.
- Policy makers charged with developing national guidelines for services would benefit from the knowledge generated and this would be reflected by well-informed, evidence-based guidance on the relative value of different service configurations.
- While not a clinical trial, this study offers insights into the cost-effectiveness of OPAT services, and provides evidence on which decisions relating to the conduct of any future randomised controlled trial could be based.

## Introduction

To meet the challenges of delivering quality healthcare, health systems must continue to develop programmes that deliver safe, high-quality, cost-effective patient care.^1^ Delivery of intravenous antibiotics to patients outside a hospital setting—often termed outpatient parenteral antimicrobial therapy (OPAT)—was first described on a small scale in the 1970s in North America. By the end of the 1990s, an estimated quarter of a million patients annually were receiving intravenous antibiotics on an outpatient basis due to a range of factors, including cost savings, patient preference, better intravenous devices, the introduction of antimicrobial agents which only needed administration once or twice a day and the introduction of new OPAT services.^2^ A wide variety of infections have been treated through this system, in particular skin and soft tissue infections,^3^ but also infections such as osteomyelitis,^4^ ^5^ bacteraemia^6^ and endocarditis.^6^ Although widely accepted as the standard of care in countries such as the USA and Australia, such services are largely limited to patients with appropriate health insurance cover.^7^

OPAT services in the UK started in the 1990s, and were limited to specialist centres, but services have recently began to expand, as the benefits to patients and the healthcare system were recognised.^2^ Several countries, including the UK have developed national guidelines (standards of care) for OPAT services to act as a benchmark for clinical monitoring and quality.^2^ ^8^ ^9^ To support the development of such services in the UK, the British Society of Antimicrobial Chemotherapy (BSAC) have sponsored the development of a toolkit which clinicians can use to develop a business case for their service. This toolkit contains SWOT analyses for each service type, but does not favour any particular model of service design.^2^ Indeed, there has been no robust examination comparing the cost-effectiveness of each service type, or any systematic examination of patient preferences for services on which to base any business case decision. Further barriers to the development of OPAT services are a lack of any specific funding system for this in the National Health Service (NHS), combined with the complexity of existing charging mechanisms.^10^

### Clinical practice in the UK

Nevertheless, in recent years, OPAT services have developed in some areas of the UK both in the NHS and private sectors, largely in response to local pressures in combination with healthcare staff initiatives.^11^ This has led to many service variations using different heathcare professionals which can be grouped into four main categories: (1) daily attendance at a hospital outpatient facility (or community-based clinic); (2) self-administration or carer administration in the patient's own home; (3) provided by community nurses with general skills, for example, district nurses in the patient's own home; (4) provided by specialist nurses, for example, NHS or private nursing teams, in the patient's own home.

## Methods and analysis

### Study objectives

- Evaluation of the existing evidence of efficacy, safety and cost-effectiveness of different intravenous antibiotic services.
- Assessment of current OPAT provision by the NHS by establishing reasons for current service configuration and identifying barriers to service provision.
- Economic modelling of the different delivery systems to evaluate their cost-effectiveness in both short-term and longer term infection patient groups.
- Determination of patient preferences for different community intravenous antibiotic service attributes through a discrete choice experiment (DCE).
- Hold an expert panel to agree on what may constitute the optimal service model(s) of community intravenous antibiotics delivery and how future clinical trials should be designed to test these services.

### Design

The study will use a mixed methods approach, combining qualitative and quantitative methodologies in five interconnected work streams. A systematic review of the published research on the safety, efficacy and cost-effectiveness of intravenous antibiotic delivery services; a qualitative study undertaking semistructured telephone interviews with 25–30 healthcare professionals managing intravenous antibiotic services in England exploring models of service offered and any perceived barriers to future development; an economic model to estimate the comparative value of four different community intravenous antibiotic services (described above) and allow comparisons of the expected costs and benefits of each service for both short-term and longer term infection patient groups. We will model the costs and benefits of running several services concurrently and providing patients with choices. Determining patient preferences using a DCE will inform which aspects of treatment are important to patients and which they would prefer in the future. Patients will be sampled from those with short-term infections having less than a week of treatment (eg, cellulitis) and those with deeper infections requiring longer treatment (eg, bone infections). The development of the DCE will involve semistructured interviews with patients and will incorporate any relevant literature. Lastly, an expert panel with a range of stakeholders to agree which service models may constitute the optimal service model(s) of community intravenous antibiotics delivery, and which are worthy of testing in clinical trials and to discuss trial design issues.

### Setting

The research will be conducted in seven acute trusts; four in West Yorkshire, one in East Yorkshire, one in South Yorkshire and one in Oxford.

### Systematic review

The aim of the review is to evaluate the existing evidence of efficacy, safety and cost-effectiveness of different intravenous antibiotic services.

There are no existing relevant systematic reviews of this topic, and this work is not an update of an existing systematic review.

Questions to be considered:

- What is the most clinically effective model of delivering intravenous antibiotics in the community?
- What is the most cost-effective model of delivering intravenous antibiotics in the community?
- What is the most appropriate model for delivering intravenous antibiotics in the community in terms of patient safety?
- Is community delivery of intravenous antibiotics acceptable to patients and care providers?

#### Searches

Two searches will be run to identify (A) studies of intravenous antibiotics and known models of care, (B) reviews of intravenous antibiotics in cellulitis or cystic fibrosis. The aim of search B is to retrieve papers mentioning models of care that we were unaware of when identifying terms for search A. A range of information sources will be searched: Bibliographic databases: MEDLINE, Medline in process, EMBASE, CINAHL, International Pharmaceutical Abstracts. The Cochrane Library, Cochrane Library evidence resources: Cochrane Database of Systematic Reviews, Cochrane Central Register of Controlled Trials. Economics resources: NHS Economic Evaluation Database, Research Papers in Economics (RePEc), Tufts Cost-Effectiveness Analysis (CEA) Registry, Health Business Elite (HBE).

Supplementary searches of Web of Science Proceedings, the Health Information Management Consortium (HMIC), International Pharmaceutical Abstracts and the website of the British Society for Antimicrobial Chemotherapy will be conducted to provide relevant unpublished work. In addition, the reference lists of included studies will be reviewed for potentially relevant papers. A sample search strategy is detailed in appendix A. The period under review is 1993–2013 (with updates planned before the submission of the final report). No language restrictions will be applied.

#### Criteria for selection of studies

Studies will be eligible for inclusion if the participants are individuals or groups of adult patients or care providers *and* they evaluate the clinical effectiveness or cost-effectiveness of an OPAT model, *or* they describe or evaluate patient safety issues associated with OPAT, *or* they consider the acceptability of OPAT from the perspective of the patient receiving treatment or the practitioner delivering care. Any form of intravenous delivery system will be included (eg, infusion or bolus).

Studies will be excluded if they make reference to clinical effectiveness but do not report on specific patient outcomes. Similarly, studies that consider costs related to a model of delivery without considering patient benefit alongside these, and studies making reference to costs and benefits without reporting specific cost-effectiveness data (eg, cost per quality-adjusted life year (QALY)) will also be excluded. Studies that include children will be reviewed but excluded if they do not report outcomes for adult patients separately. Studies involving multiple routes of delivery of antibiotics will be reviewed but will be excluded if they do not report outcomes for patients receiving intravenous treatment separately.

The outcomes of interest are clinical effectiveness (cure or improvement, duration of treatment), cost-effectiveness (cost, benefit), safety (complications, adverse events, hospital admission), patient acceptability and provider acceptability.

Both experimental (randomised controlled trials, controlled clinical trials, controlled before and after studies) and non-experimental (case–control, cohort, cross-sectional, other observational) studies will be included. Quantitative, qualitative and mixed-methodology studies will be included.

The review and synthesis of data will be undertaken in accordance with the Centre for Reviews and Dissemination guidelines for systematic reviews.^12^ A protocol of the review was produced and added to the PROSPERO database (CRD ref: 4201300637 4).

#### Data extraction

Abstracts of all identified studies will be screened for inclusion by one reviewer with a random selection (20%) independently screened by a second reviewer. Potentially relevant studies will then be independently assessed by two reviewers to determine whether they meet the inclusion criteria. Differences of opinion will be discussed until a consensus is reached; the opinion of a third reviewer will be sought where necessary.

Data extraction will be carried out by one reviewer using a standardised proforma. Extracted data will include citation details, study purpose, design, location, setting, duration, population details and clinical characteristics (reason for antibiotic treatment), models of care (outpatient, self-administration, general nurse, specialist nurse or other), topic area (clinical effectiveness, cost-effectiveness, safety, acceptability), type of antibiotic, route of delivery, treatment dose, outcome measures (if relevant), follow-up and key findings.

#### Quality assessment

Quality assessment will be carried out by one reviewer. The Cochrane Risk of Bias assessment tool will be used for experimental studies, and the Newcastle-Ottawa scales for cohort and case–control studies.^12^ ^13^ We will use critical interpretive synthesis developed by Dixon-Woods *et al*^14^ to review and analyse any qualitative literature. The quality of studies reporting economic evaluations will be assessed using the Drummond *et al*^15^ checklist. Studies will be selected by two researchers working independently with inconsistencies resolved following discussion.

Studies at risk of bias will not be excluded from the review, but an appraisal of the strength of existing evidence will be reported, and the review findings interpreted in light of this.

#### Evidence synthesis

The main characteristics of included studies and findings relating to clinical effectiveness, cost-effectiveness, patient safety and acceptability, and study quality will be summarised in narrative and tabular form. We anticipate that there will be substantial heterogeneity between included studies, and as such do not plan to pool data for meta-analysis. The synthesised review data will provide values for use in the economic model, DCE and evidence for consideration by the expert panel.

### Qualitative study

The aim of this work package is to assess current NHS OPAT provision by establishing reasons for different service configurations and identifying barriers to service provision.

#### Design: qualitative telephone interviews

Initially, a brief electronic or telephone survey will be administered to infection unit managers and/or infection specialists to establish types of services available in England. This will be used to identify potential interviewees.

#### Sampling

Purposive sampling via a sampling matrix will recruit participants with different experiences of delivering OPAT services. A sample of 25–30 infection specialists and/or unit managers will be recruited. This will ensure a detailed and comprehensive range of perspectives and participants.

#### Data collection

Semistructured, telephone interviews will be conducted using a topic guide developed from the literature review and expert opinion (clinician co-applicants/advisors and Patient and Public Involvement (PPI) members). The researcher will probe pertinent initial responses and expand on issues raised. Interviews will be recorded and transcribed verbatim.

#### Data analysis

We will use the Framework approach^16^ to analyse the data in five stages: (1) familiarisation with the data, (2) identifying the thematic framework, (3) indexing, (4) charting, and (5) mapping and interpreting. This process enables the researcher to identify emerging themes or issues in the data. Little is known about why NHS Trusts choose to deliver specific OPAT models. Evidence generated from the literature review and input from our clinical co-applicants will be used to refine the thematic framework. The thematic analysis will be modified in the light of new data, and a process of constant comparison will be used to examine across themes and cases. The findings from the interviews will inform the modelling and DCE work streams and will be considered by the expert panel.

### Cost-effectiveness

This work package will provide an economic model of the different delivery systems to evaluate their cost-effectiveness in both short-term and longer term infection patients groups.

A decision-analytic model will be developed to estimate the cost-effectiveness of OPAT services. Published best practice modelling guidelines will be applied.^17^ The analysis will conform to the reference case set out by the National Institute for Health and Care Excellence (NICE)^18^ using a cost-utility analysis (CUA) between service models and effectiveness measured by QALYs. It is possible that the relative benefits of the service models may not register in terms of QALY gains (especially for short-term infections where treatment may last less than 1 week). Therefore, we will also conduct a cost-effectiveness analysis based on cost per patient successfully treated. We will take the perspectives of the healthcare provider and personal social services and of the patient/carer in separate analyses. Daily hospital treatment service will be considered ‘standard care’ for the analysis. The model structure will be informed by previous models in the area and through discussions with clinicians and patients.

We will adhere to NICE technology appraisal guidelines and subsequent Technical Support Documents produced by the NICE Decision Support Unit in identifying and selecting parameters.^19^ Data (eg, NHS resource use and costs, service model efficacy, event and risk probabilities and utility values) for the economic model will be derived from the literature review, from the DCE interviews, from retrospective retrieval of hospital and general practitioner records and from clinical experts. Patients’ use of NHS resources will be captured indirectly from medical records and directly from patient reports of service use by those participating in the DCE. Research nurses will extract data (with patient consent) on treatments received, effectiveness, duration and location, treatment delivery systems, additional health services used or visits required and adverse events. We aim to extract a minimum of 400 records across short-term and long-term infection patients and across services (ie, n=50 per infection type per service model). All data will be anonymised. If we have insufficient data on particular parameters, we may convene a clinical expert and patient consensus panel to agree on suitable model values.

Unit costs for treatments, health service staff and resources will be obtained from the Personal Social Services Research Unit (PSSRU), the British National Formulary (BNF) and NHS Reference cost database. Health state utility values for the CUA will be obtained from those participating in the DCE completing an EQ-5D^20^ questionnaire, which is NICE's preferred source of utility values. The EQ-5D is a simple, five-item questionnaire which provides utility values based on a UK general population-derived tariff.

#### Data analysis

The CUA will compare the three models of community intravenous antibiotic service delivery to daily hospital attendance (considered ‘standard care’). The Markov decision model will be developed to describe the patient pathway and potential outcomes and provide estimations of expected costs and benefits for the service models. The results will be presented as expected incremental cost-effectiveness ratio, expected net benefit (assuming λ is equal to the NICE QALY threshold of £20 000) and a cost-effectiveness acceptability curve.^21^ QALYs will be based on EQ-5D utility values although estimates for other health states that may be required in the model (eg, for hospitalisation or hospital-acquired infections) may come from direct values found in the literature.

Deterministic one-way, multiway and scenario sensitivity analyses will explore the sensitivity of the results to the modelling assumptions and parameter values selected. Probabilistic sensitivity analysis via Monte Carlo simulations will be undertaken to test the robustness of the results to parameter uncertainty. We will use the value of information framework to explicitly quantify the implications of decision uncertainty. We will calculate the expected value of perfect information^22^ to help determine the value of, and priorities for, future research and the expected value of sample information to design efficient future research. Recently proposed regression methods for achieving this will be employed.^23^ This information will be used by the team and clinical trial statisticians to plan any future clinical trials.

### Discrete choice experiment

This work package will determine patient preferences for different community intravenous antibiotic service attributes through the use of a DCE.

The quantitative study of patient preference choices involves the use of discrete choice models, which are mathematical structures belonging to the family of random utility models (for a comprehensive review see Train^24^). A substantial number of studies in health economics now make use of choice modelling techniques^25^ and our methodology will follow best practice established in Lanscar and Louviere.^26^ Attribute development will be conducted in two phases using recommendations by Coast *et al*^27^ followed by the development and piloting of the DCE interview outlined by Willis.^28^ Information from the literature review and record retrieval (eg, on treatment effectiveness) will also inform survey content. Our patient advisory group (PAG) will be closely involved with this work package, providing input into the development of study materials (eg, DCE items) and interpretation of interview data. They will also provide a patient perspective on the interpretation of the resulting models.

#### Phase 1 (attribute development)

Design: We will undertake a qualitative exploration of patient's experiences of receiving intravenous antibiotic services and their views on the important aspects of the service. The use of primary qualitative data to inform the development of DCEs is relatively recent; therefore, we will follow guidance developed by Coast *et al*.^27^

Sample: Adults (n=30–50) who have experienced community intravenous antibiotic treatment will be purposively selected using a sampling matrix ensuring a range of intravenous antibiotic services, treatment lengths and diverse backgrounds are represented.

##### Data collection

Focus groups will be offered to participants to present their ideas and experiences, and to generate new ideas. To maximise recruitment, interviews will be offered to those unable/reluctant to attend a focus group. A topic guide will be developed from the literature to guide discussions, and all sessions will be facilitated by experienced researchers. All session will be recorded (with consent) and transcribed verbatim.

##### Data analysis

The principle approach will be content analysis, with data analysed for patterns and themes, to develop categories and subcategories of attributes to generate a comprehensive set of attributes. Data will be analysed iteratively using constant comparative methods^29^ involving close reading of the data to identify words that capture thoughts or concepts. The results of the analysis will be used to construct the attributes and levels for the DCE scenarios.

#### Phase 2 (refinement of terms)

After further refinement, we will pilot the DCE using a ‘think aloud’ cognitive interviewing technique^28^ with a ‘naive’ sample of participants (n=30) to test understanding and identify problems.

#### Phase 3 DCE

Sample: Participants will be recruited from the sites providing each of the services under investigation (described above). Patients will be eligible if they are currently receiving intravenous antibiotics in the community, as an outpatient, or have previously received such a course of intravenous antibiotics within the past 24 months (identified retrospectively from records).

##### Sample size

The sample sizes for DCEs depend on the number of choice tasks completed by respondents and the number of attributes and levels presented in each choice. As the first stage of the study is the development of the DCE, we have used estimates from previous work which would suggest that 200 participants with short-term and longer term infections should be sufficient to allow determination of robust preference data.^30^ We will use advanced survey design techniques that maximise the potential for trading between attributes to enhance the data quality.^31^

##### Data collection

The interviews will be carried out by the researcher using a laptop to present the choices to respondents on a one-to-one basis.

##### DCE questionnaire

We envisage presenting each respondent with 8–10 different choice scenarios. The planned pilot survey will allow us to determine the most appropriate survey design.

##### Data analysis

Data will be analysed using advanced mixture models allowing us to accommodate the expected high level of heterogeneity in sensitivities/preferences across individual patients.^32^ We will seek to explain this heterogeneity by linking sensitivities to patient characteristics, and the use of constructs that allow us to explicitly incorporate underlying attitudes.^33^ Any remaining unexplained heterogeneity will be accommodated in a random manner. Hess *et al*^34^ ^35^ have shown that an appropriate approach to the various types of heterogeneity is likely to provide more robust overall measures of sensitivities. The attitudinal data will also mitigate the effects of any strategic bias that may arise in the data.

### Expert panel

The final work package comprises an expert panel to agree on what may constitute the optimal service model(s) of community intravenous antibiotics delivery and how future clinical trials should be designed to test these services.

The expert panel will consider the evidence generated from the literature review, economic modelling, DCE and survey of current service provision. A consensus will be sought on what is likely to represent optimal community intravenous antibiotic therapy for the two patient groups (long-term and short-term intravenous antibiotic patients). Membership of the panel will consist of 2–4 service user representatives (including members of our PAG) together with healthcare staff currently involved in intravenous antibiotic delivery, pharmacists, GPs, NHS commissioners, health economists and a clinical trials statistician.

#### Data collection and analysis

The expert panel will consider which service delivery models and which particular configuration of these models warrant full evaluation in a clinical trial. The panel will consider the design of future clinical trials, identifying ways to overcome potential barriers to service provision and formulate a plan of future research priorities.

The proposed research will address significant gaps in knowledge about the clinical and cost-effectiveness of different intravenous antibiotic services; identify which services patients prefer and which aspects of the services are most important to them. Since the services available to patients are likely to have different costs, effects and risks, it is essential to understand what patients consider most important in the care they receive and what trade-offs they are willing to make. The optimal delivery of OPAT may mean offering patients a choice between several services concurrently which has consequences for future planning and resourcing. The panel will be asked to consider these options.

### Consent

Patients with experience of community intravenous antibiotic treatment will be approached by the research nurses either while they are still under the care of the hospital or retrospectively when then have completed treatment. All eligible patients will be given a patient information leaflet to consider and if they are willing to participate they will be consented and registered.

### Confidentiality

Monitoring and extraction of data from patient records will be carried out by the research nurses with the full consent of the patient and anonymised. Data will be handled in accordance with the 1998 Data Protection Act at all times. Patients and staff members who consent to be interviewed or to participate in focus groups will be assured of the confidentiality and secure handling of the recordings and transcripts.

### Dissemination policy

An end of project national dissemination meeting is planned for NHS commissioners and clinicians to present the findings of the studies and the recommendations of the expert panel. A lay summary of the project will be produced for study participants. Findings will be presented at relevant conferences such as the National OPAT conference and Federation of Infections Societies conference. The chief investigator and co-applicants will be named as authors on main publications, and an appropriate first author agreed through discussion. Other key individuals will be included as authors or contributors as appropriate, at the discretion of the Senior Management Group (SMG). Any disputes relating to authorship will be resolved by the Steering Committee.

The Chair and Independent members of the Steering Committee will be acknowledged, but will not qualify for full authorship, in order to maintain their independence. Individual collaborators must not publish data concerning their participants’ which are directly relevant to the questions posed in the study until the main results of the study have been published.

### Conclusion

The full potential of OPAT has not yet been realised in the UK as there is patchy implementation and significant variation in services geographically. There is a paucity of information on which the NHS can base decisions regarding the design, supply and commissioning of such services and on which national guidance developers can base recommendations for best practice. OPAT services have the potential to generate significant cost savings for the NHS and deliver greater patient satisfaction. They may also contribute to the delivery of key healthcare strategies and directives such as ‘Equity and Excellence: Liberating the NHS’ (2010), ‘Creating a Patient-led NHS’ (2005) and ‘Your Health, Your Care, Your Say’ (2006).
